# An updated analysis of large language model performance on ophthalmology speciality examinations

**DOI:** 10.1038/s41433-026-04262-1

**Published:** 2026-01-30

**Authors:** Abdallah S. H. Abbas, Ariel Yuhan Ong, Fares Antaki, Haseeb N. Akhtar, Mai Shehab, Pearse A. Keane

**Affiliations:** 1 Institute of Ophthalmology, University College London, London, UK; 2 Moorfields Eye Hospital, London, UK; 3 NIHR Biomedical Research Centre, Moorfields Eye Hospital NHS Foundation Trust, London, UK; 4 Cole Eye Institute, Cleveland Clinic, Cleveland, OH USA; 5 Department of Ophthalmology, Centre Hospitalier de l’Universite de Montreal, Montreal, QC Canada; 6 The CHUM School of Artificial Intelligence in Healthcare (SAIH), Centre Hospitalier de l’Université de Montréal (CHUM), Montreal, QC Canada; 7 Mid & South Essex NHS Trust, Essex, UK

**Keywords:** Outcomes research, Health services

Large language models (LLMs) have evolved rapidly since their public release in November 2022, with recent iterations integrating multi-modal and advanced reasoning capabilities. As their deployment within healthcare expands, rigorous benchmarking is essential. In 2023, Raimondi et al. evaluated LLM performance on the Fellowship of Royal College of Ophthalmologists (FRCOphth) exams [1], the standard for independent practice in the United Kingdom. This study reports an updated benchmark of current-generation models, supplemented by a qualitative error analysis to examine cases of model failure.

Multiple choice questions were sourced from mock examinations published by the Royal College of Ophthalmologists [2, 3]. The official answer provided was used as the ground truth. This was the same dataset used by Raimondi et al. in 2023. Fifty FRCOphth Part One (Basic Sciences) and forty-three Part Two (Clinical Application) questions were included.

Six models were evaluated: ChatGPT 4o and 5 (OpenAI), Gemini 2.5 Pro (Google), Claude Sonnet 4.0 (Anthropic), Deepseek-V3 (Deepseek) and Grok 3 (xAI). Models were tested in July 2025 except ChatGPT 5 (September 2025). Models were evaluated via the official chat interfaces in default configurations, without adjusting reasoning duration. Zero-shot prompting was used with questions presented as they appear in the sample paper, with one correct answer and three distractors. No system instruction was given.

LLM model performance in 2025 was compared with 2023 using the Mann–Whitney *U* test, after exclusion of one image-based question to ensure matched datasets. The Hodges-Lehmann estimator was used to quantify the median difference between 2023 and 2025 models, reported with 95% confidence intervals. Pairwise Cohen’s Kappa (*κ*) was used to assess inter-model agreement. Additionally, McNemar’s test was used to compare the performance of ChatGPT 4o and ChatGPT 5. All analyses were conducted using Python (version 3.12.12), and results were considered statistically significant at *p* < 0.05.

An error analysis was performed on questions where all models reached a consensus on the same incorrect option. Consultants with domain-specific expertise, initially masked to model outputs, independently answered each question before evaluating the models’ rationale. Furthermore, incorrect responses to the image-based question were analysed to identify specific reasoning failure mechanisms.

Gemini 2.5 Pro achieved the highest accuracy in both examinations (Part One: 98.0%; Part Two: 90.7%; Fig. 1). The remaining models performed as follows**:** Claude Sonnet 4.0 (94.0%; 74.4%), Grok 3 (92.0%; 88.4%), ChatGPT 4o (90.0%; 88.4%), ChatGPT 5 (90.0%; 86.0%), and DeepSeek-V3 (82.0%; 83.7%).

**Fig. 1:**
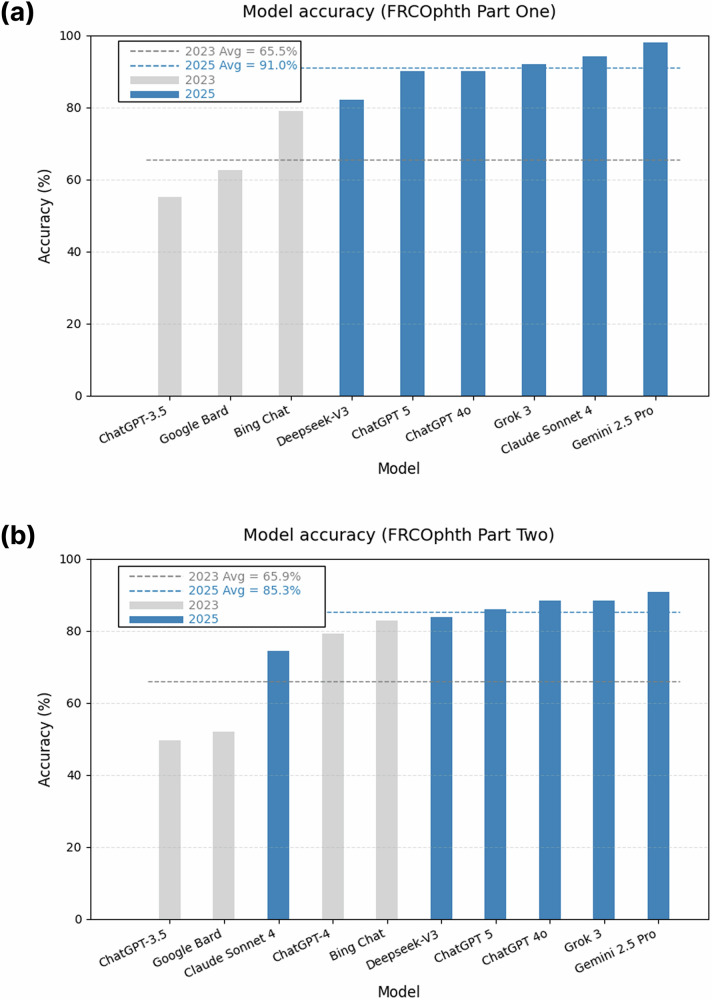
Accuracy (%) of LLMs on FRCOphth examinations. **a** Part One (Basic Sciences). **b** Part Two (Clinical Reasoning). Blue bars represent 2025 models; grey bars show 2023 results from Raimondi et al. Mean accuracy for each year is indicated by the dashed line.

Compared with the 2023 models [1], the 2025 cohort showed significant improvements in accuracy for both Part One (*p* = 0.028) and Part Two (*p* = 0.042). The Hodges–Lehmann estimator indicated a median increase of 27.2 percentage points in Part One (95% CI [2.7, 40.5]) and 17.1 percentage points in Part Two (95% CI [0.8, 38.8]). Performance between ChatGPT 4o and ChatGPT 5 was near-identical (McNemar *p* = 1.00), with high agreement between the two models in both Part One (*κ* = 0.89) and Part Two (*κ* = 0.94). Performance breakdowns by sub-topic and full pairwise κ-matrices are provided in the Supplementary Materials.

Inter-model consensus was reached on the same incorrect answer in two instances (Supplementary Materials). The first queried the most likely feature associated with symptomatic retinal dialysis. All models selected ‘other signs of blunt ocular trauma’, whereas the ground truth was ‘macular detachment’. Expert vitreoretinal consultant review favoured the LLM consensus, citing that those presenting with a dialysis may have a field defect but usually present prior to macular detachment.

The second question involved a patient with a family history of glaucoma, bilateral intra-ocular pressures of 25 mmHg, optic atrophy and reduced visual acuity. When asked for the next step in managing this case, the models unanimously opted for pharmacological intervention with latanoprost, while the ground truth was MRI brain and orbit. Two consultant ophthalmologists sub-specialising in glaucoma characterised the LLM reasoning as superficial. The models failed to integrate atypical features necessitating neuroimaging to exclude compressive pathology. Specifically, the presence of optic atrophy with reduced visual acuity, the absence of correlating RNFL damage, and the asymmetry of visual function despite symmetric intraocular pressure.

A mean accuracy of 66.7% was achieved for the single multimodal task involving an optical cross transposition. Deepseek-V3 successfully derived the correct prescription within its reasoning trace but failed to select the corresponding multiple-choice selection. In contrast, Claude Sonnet 4 exhibited a failure in axis alignment, suggesting a deficit in spatial reasoning.

We have shown current generation LLMs to significantly outperform previous models on postgraduate ophthalmology examinations. Notably, most models now exceed the top human candidate score of 85.0% in Part One and 84.2% in Part Two, based on the most recent official examination reports [4, 5]. Gemini 2.5 Pro achieved the highest accuracy (98.0% Part One; 90.7% Part Two), exceeding the previous top performer (Bing Chat, 78.9% and 82.9% respectively) [1].

Our error analysis revealed an instance where models exhibited premature diagnostic closure, relying on superficial pattern-matching rather than systematically excluding alternative aetiologies. Reliance on such outputs presents a tangible safety risk, as the failure to integrate discordant negative findings (e.g., lack of corresponding RNFL damage) led to the omission of essential neuroimaging to rule out compressive pathology.

Multimodal capabilities now permit the assessment of visual reasoning, however this dataset was restricted to a single image-based task. To evaluate true clinical utility, future benchmarking requires a more diverse dataset encompassing different diagnostic modalities.

There remains a potential risk of data contamination, where public examinations seen during training lead to hyper-inflated performance. However, given the closed nature of pre-training corpora for frontier models, this limitation applies broadly to all studies using public benchmarks.

Use of LLMs is likely to become ubiquitous in future clinical practice, though at this stage superior exam performance is compromised by the persistence of safety-critical reasoning deficits. Future research should therefore seek to evaluate ‘clinician-in-the-loop’ workflows, ensuring human oversight while harnessing the model’s strengths.

Supplemental materials are available at Eye’s website.

## Supplementary information

Supplementary Materials

## Data Availability

The datasets generated and analysed during the current study are available from the corresponding author on reasonable request. FRCOphth Part 1 and Part 2 Written examination sample questions are freely available online.
