# Supplementary material for: An updated analysis of large language model performance on ophthalmology speciality examinations
Source: Eye (Lond). 2026 Jan 30;40(5):572–4. doi: 10.1038/s41433-026-04262-1 (PMC13013908; doi:10.1038/s41433-026-04262-1)
Supplement: Supplementary file 1 — Supplementary Materials [file 41433_2026_4262_MOESM1_ESM.docx]

**Supplementary Materials**

**Supplementary Table 1**. FRCOphth Mock Examination Questions included in error analysis

| **Question** | **Ground truth** | **LLMs** | **Expert** |
| --- | --- | --- | --- |
| Which one of the following statements is MOST likely to be correct for a patient presenting with a symptomatic retinal dialysis?  A. Other signs of blunt ocular trauma are usually present  B. The duration of the retinal detachment is generally a week or less  C. The macula is likely to be detached  D. The site of the dialysis is most commonly infero-nasal | C | A | A |
| A 60 year old woman with a family history of glaucoma is referred by the optometrist with deteriorating vision and an arcuate field defect in the left eye. The corrected visual acuity is 6/9 right eye and 6/18 left eye. The media are clear. The intraocular pressure is 25mm Hg in both eyes with corneal thickness of 580 microns. The left eye has mild optic atrophy and peripapillary choroidal atrophy.  What would be the MOST appropriate next line of management?  A. MRI scan of orbit and brain  B. Review in 3-4 months with further visual fields  C. Start treatment with latanoprost eye drops  D. Visual evoked potentials | A | C | A |

**Supplementary Figure 1.** Model accuracy by topic (a) FRCOphth Part One (b) FRCOphth Part Two.

**
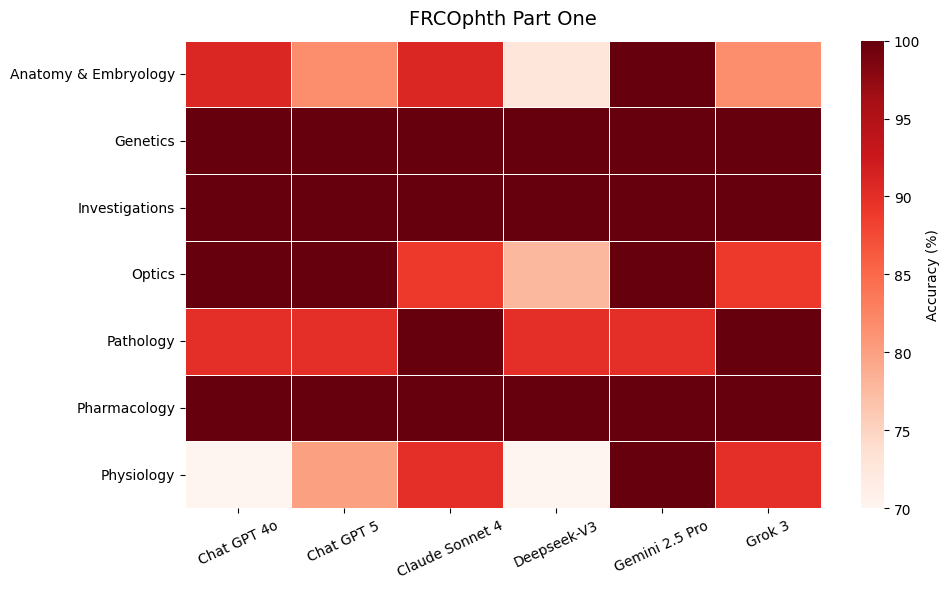
**

**
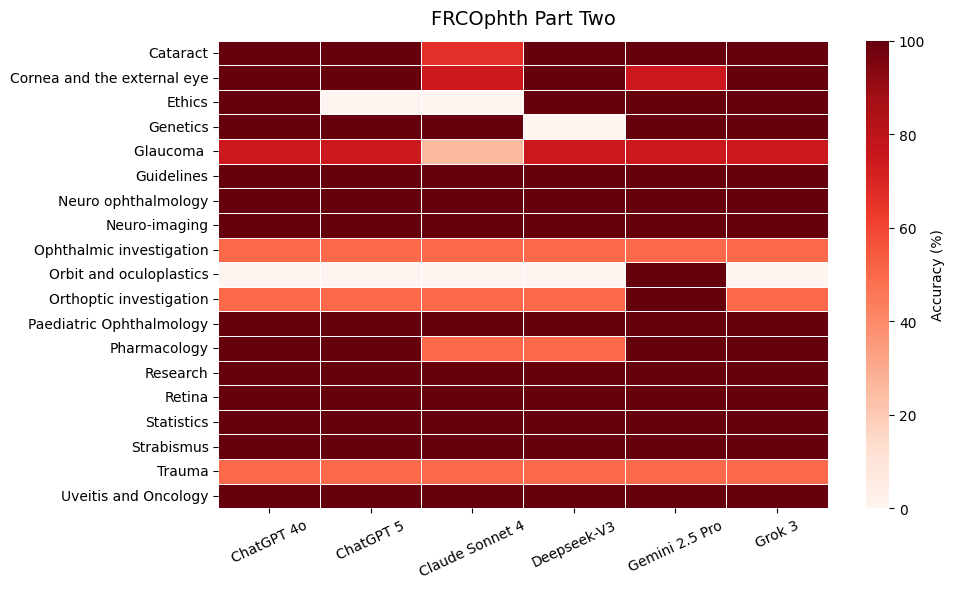
**

**Supplementary Figure 2.** Inter-Model Agreement Using Pairwise Cohen’s Kappa (a) FRCOphth Part One (b) FRCOphth Part Two.

**
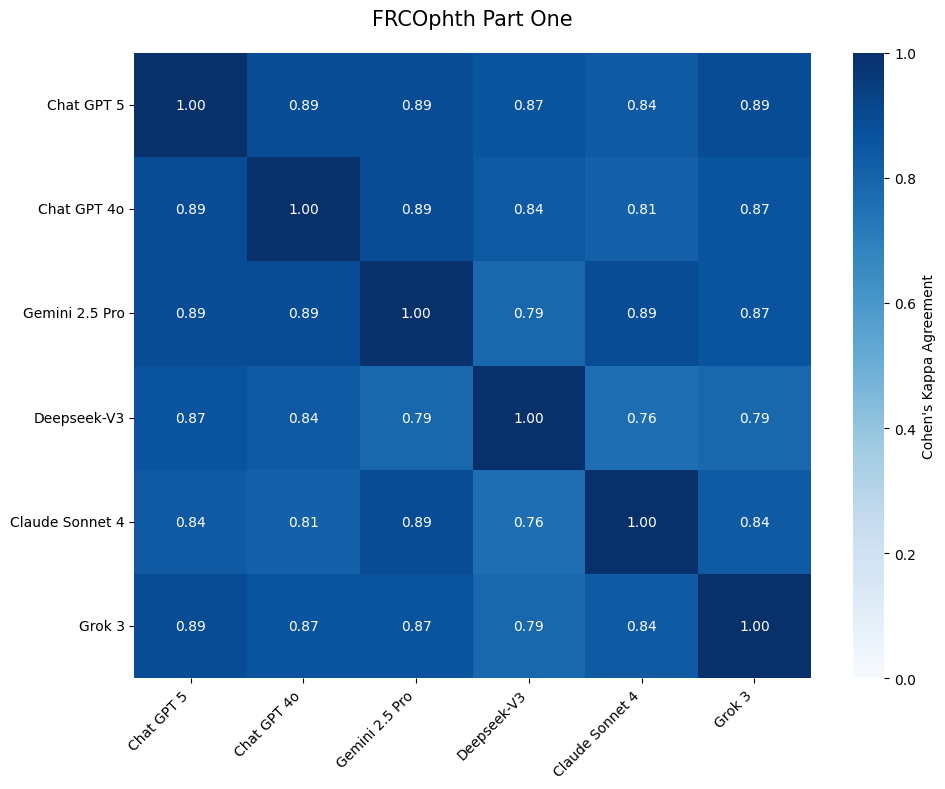
**

**
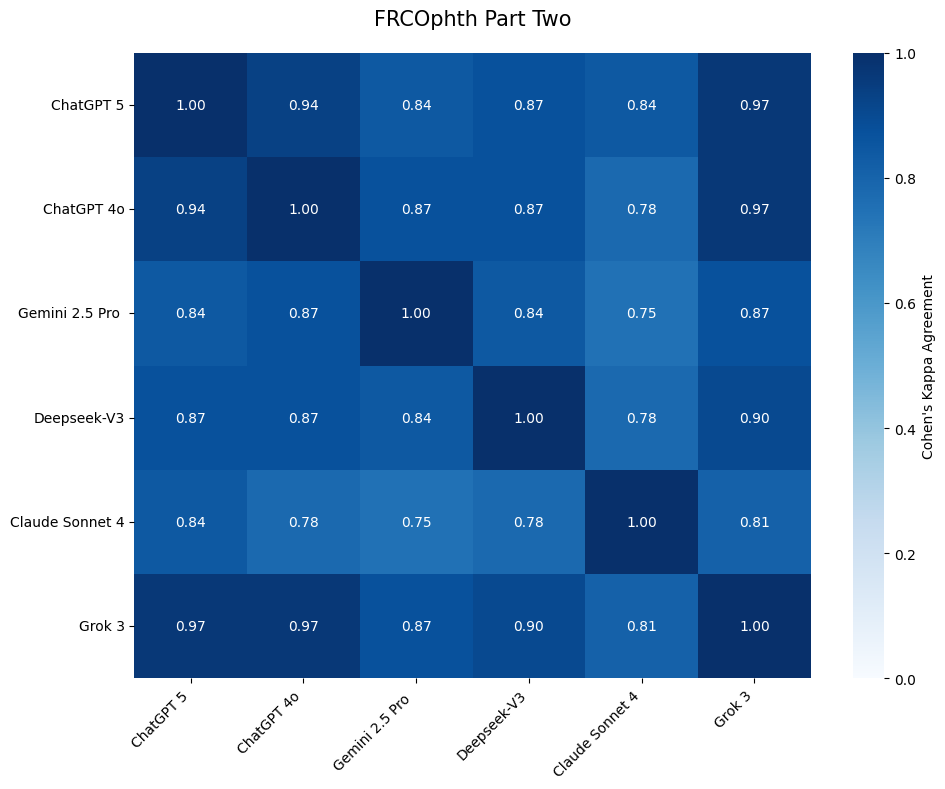
**
